# 8-[(Hydrazinyl­idene)meth­yl]-4-methyl-2-oxo-2*H*-chromen-7-yl 4-methyl­benzene­sulfonate

**DOI:** 10.1107/S1600536810054620

**Published:** 2011-01-12

**Authors:** H. Yuvaraj, D. Gayathri, Rajesh G. Kalkhambkar, G. M. Kulkarni, Rajendra M. Bapset

**Affiliations:** a School of Display and Chemical Engineering, Yeungnam University, Gyeongsan, Gyeoungbuk 712-749, Republic of Korea; b Institute of Structural Biology and Biophysics-2: Molecular Biophysics, Research Centre Jülich, D-52425 Jülich, Germany; c Department of Chemistry, Karnatak University’s Karnatak Science College, Dharwad 580 001, Karnataka, India; d Department of Chemistry, B. K. College, Belgaum 590 001, Karnataka, India

## Abstract

In the title compound, C_18_H_16_N_2_O_5_S, the coumarin ring system is nearly planar, with a maximum out-of-plane deviation of 0.078 (1) Å (r.m.s. deviation = 0.046 Å). The dihedral angle between the coumarin ring system and the toluene ring (r.m.s. deviation = 0.004 Å) is 2.77 (1)°. The crystal packing is stabilized by C—H⋯O and N—H⋯O inter­molecular hydrogen bonds generating *C*(8), *C*(9) and *C*(11) chains and *R*_2_^2^(14), *R*_2_^2^(23) and *R*_4_^3^(13) ring graph sets.

## Related literature

For the biological activity of coumarins, see: Kulkarni *et al.* (2006 ▶); Kalkhambkar *et al.* (2008 ▶); Laakso *et al.* (1994 ▶); Nofal *et al.* (2000 ▶). For related structures, see: Kokila *et al.* (1995 ▶); Vasudevan *et al.* (1990 ▶). For graph-set analysis of hydrogen-bond patterns, see: Bernstein *et al.* (1995 ▶).
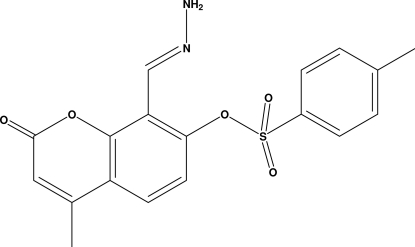


## Experimental

### 

#### Crystal data

C_18_H_16_N_2_O_5_S
*M* *_r_* = 372.39
Monoclinic, 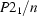

*a* = 9.1947 (3) Å
*b* = 16.1867 (4) Å
*c* = 11.6538 (3) Å
β = 99.670 (1)°
*V* = 1709.81 (8) Å^3^
*Z* = 4
Mo *K*α radiation
μ = 0.22 mm^−1^
*T* = 293 K
0.2 × 0.19 × 0.19 mm

#### Data collection

Bruker SMART CCD area-detector diffractometer
26334 measured reflections
4260 independent reflections
3285 reflections with *I* > 2σ(*I*)
*R*_int_ = 0.033

#### Refinement

*R*[*F* ^2^ > 2σ(*F* ^2^)] = 0.040
*wR*(*F* ^2^) = 0.116
*S* = 1.04
4260 reflections
237 parameters
H-atom parameters constrained
Δρ_max_ = 0.35 e Å^−3^
Δρ_min_ = −0.40 e Å^−3^

### 

Data collection: *SMART* (Bruker, 2001 ▶); cell refinement: *SAINT* (Bruker, 2001 ▶); data reduction: *SAINT*; program(s) used to solve structure: *SHELXS97* (Sheldrick, 2008 ▶); program(s) used to refine structure: *SHELXL97* (Sheldrick, 2008 ▶); molecular graphics: *PLATON* (Spek, 2009 ▶); software used to prepare material for publication: *WinGX* (Farrugia, 1999 ▶) and *PARST* (Nardelli, 1995 ▶).

## Supplementary Material

Crystal structure: contains datablocks I, global. DOI: 10.1107/S1600536810054620/si2319sup1.cif

Structure factors: contains datablocks I. DOI: 10.1107/S1600536810054620/si2319Isup2.hkl

Additional supplementary materials:  crystallographic information; 3D view; checkCIF report

## Figures and Tables

**Table 1:** Hydrogen-bond geometry (Å, °)

*D*—H⋯*A*	*D*—H	H⋯*A*	*D*⋯*A*	*D*—H⋯*A*
N2—H2*A*⋯O5^i^	0.86	2.59	3.303 (2)	141
N2—H2*B*⋯O1^ii^	0.86	2.27	3.045 (2)	150
C10—H10⋯O4^iii^	0.93	2.55	3.469 (2)	169
C16—H16*A*⋯O4^ii^	0.96	2.55	3.405 (3)	149
C18—H18⋯O1^iv^	0.93	2.53	3.166 (2)	126

Symmetry codes: (i) 
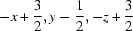
; (ii) 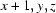; (iii) 
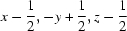
; (iv) 
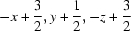
.

**Fig. 1.**
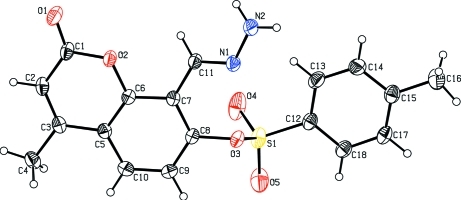
The molecular structure of title compound, showing 30% probability displacement ellipsoids.

**Fig. 2.**
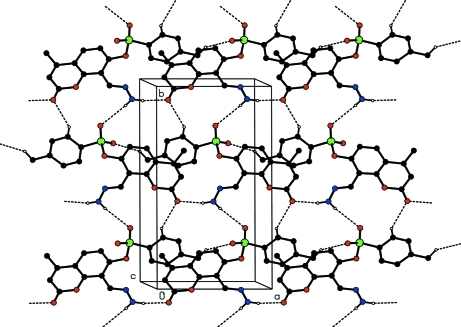
The molecular packing of (I) showing the ring graph sets generated by N—H···O and C—H···O intermolecular interactions. For clarity, hydrogen atoms which are not involved in hydrogen bonding are omitted.

**Fig. 3.**
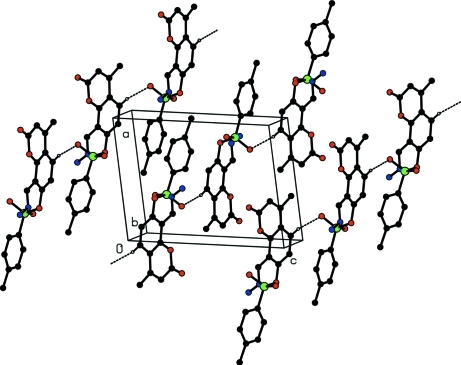
The molecular packing of (I) showing C10—H10···O4 intermolecular interaction. For clarity, hydrogen atoms which are not involved in hydrogen bonding are omitted.

## supplementary crystallographic information

### Comment

Coumarins represent a group of naturally occurring lactones, whose potential as anti-inflammatory, anti-microbial, anticancer and protease inhibiting agents has recently been reviewed (Kulkarni *et al.*, 2006). Coumarin derivatives with various substituents at the C-4 position with their biological activities have been reported from our laboratory (Kalkhambkar *et al.*, 2008). Many natural coumarins are reported for their wide range of biological and antitumor properties (Nofal *et al.*, 2000). Solid-state conformations of 4-aryloxymethyl and 4-aryl aminomethyl coumarins have been found to be significantly different. The former exhibits a centro-symmetric nature (Vasudevan *et al.*, 1990) in the solid state, whereas the latter have been found to exhibit a layer like structure stabilized by inter molecular hydrogen bonds (Kokila *et al.*, 1995). In view of biological importance of coumarin we synthesized the title compound and report here its structure.

The molecular structure of the title compound is shown in Fig.1. The coumarin ring system is nearly planar with a maximum out-of-plane deviation of 0.078 (1) Å (r.m.s. deviation = 0.046 Å). The dihedral angle between the coumarin ring system and the toluene ring (r.m.s. deviation = 0.004 Å) is 2.77 (1)°. Atoms O1 and C4 lie 0.066 (2) and 0.005 (2) Å, respectively, below the least-squares plane of the atoms (C1/C2/C3/C5/C6/O2). Atom C16 lies -0.008 (2) Å from the least-squares plane of the ring to which it is attached. Torsion angle C8—C7—C11—N1 (-15.2 (2)°) indicates slight deviation of hydrazonomethyl group from the plane of benzo-ring in coumarin moeity.

The crystal packing is stabilized by N—H···O and C—H···O intermolecular hydrogen bonds. N2—H2A···O5^i^, C18—H18···O1^iv^; N2—H2B···O1^ii^, C16—H16A···O4^ii^ generate chains of C(9), C(11) {along [010]}; C(9), C(8) {along [100]}, respectively. These intermolecular hydrogen bonds, in turn, generate *R*_2_^2^(14), *R*_2_^2^(23) and *R*_4_^3^(13) graph sets (Bernstein *et al.*, 1995) (Table 1, Fig. 2). The crystal packing is further stabilized by C10—H10···O4^iii^ intermolecular hydrogen bond generating C(8) chain along *ac* plane. The glide plane symmetry operation and translation along the *a* axis link the molecules into a three-dimensional network *via* intermolecular hydrogen bonds (Fig. 3).

### Experimental

A mixture of toluene-4-sulfonicacid-8-formyl-4-methyl-2-oxo-2*H*-chromen- 7-ylester (6 mmol), and hydrazine hydrate (6 mmol) in 20 ml of ethanol- acetic acid mixture (2:1) was refluxed on water bath for 6 h. Once the reaction was over, the excess of solvent was removed under reduced pressure and filtered the separated solids. The solids were then washed with excess of cold water, dried and crystallized from ethanol and dioxan mixture. Yield: 78%; Colorless crystalline solid (ethanol); mp 160–162 °C; *R*_f_ 0.66 (benzene); IR (KBr) cm^-1^ 3405, 1724, 1627, 1341; ^1^H NMR (CDCl_3_ + TFA) δ 2.37 (3*H*, s), 2.54 (3*H*, s), 6.47 (1*H*, s), 7.34 (2*H*, s), 7.47 (6*H*, m), 8.68 (1*H*, s); Anal.Calc.for C_18_H_16_N_2_O_5_S: C, 58.05; H, 4.33; N, 7.52; Found: C, 57.91; H, 4.20; N, 7.27.

### Refinement

All H-atoms were refined using a riding model with d(C—H) = 0.93 Å, *U*_iso_ = 1.2*U*_eq_ (C) for aromatic CH, 0.96 Å, *U*_iso_ = 1.5*U*_eq_ (C) for CH_3_ and 0.86 Å, *U*_iso_ = 1.2*U*_eq_ (N) for NH_2_ atom.

### Crystal data

**Table:**

C_18_H_16_N_2_O_5_S	*F*(000) = 776
*M**_r_* = 372.39	*D*_x_ = 1.447 Mg m^−^^3^
Monoclinic, *P*2_1_/*n*	Mo *K*α radiation, λ = 0.71073 Å
Hall symbol: -P 2yn	Cell parameters from 7131 reflections
*a* = 9.1947 (3) Å	θ = 2.2–27.3°
*b* = 16.1867 (4) Å	µ = 0.22 mm^−^^1^
*c* = 11.6538 (3) Å	*T* = 293 K
β = 99.670 (1)°	Plate, colorless
*V* = 1709.81 (8) Å^3^	0.2 × 0.19 × 0.19 mm
*Z* = 4	

### Data collection

**Table:**

Bruker SMART CCD area-detector diffractometer	3285 reflections with *I* > 2σ(*I*)
Radiation source: fine-focus sealed tube	*R*_int_ = 0.033
graphite	θ_max_ = 28.3°, θ_min_ = 2.2°
phi and ω scans	*h* = −12→12
26334 measured reflections	*k* = −21→21
4260 independent reflections	*l* = −15→15

### Refinement

**Table:**

Refinement on *F*^2^	Primary atom site location: structure-invariant direct methods
Least-squares matrix: full	Secondary atom site location: difference Fourier map
*R*[*F*^2^ > 2σ(*F*^2^)] = 0.040	Hydrogen site location: inferred from neighbouring sites
*wR*(*F*^2^) = 0.116	H-atom parameters constrained
*S* = 1.04	*w* = 1/[σ^2^(*F*_o_^2^) + (0.0548*P*)^2^ + 0.5733*P*] where *P* = (*F*_o_^2^ + 2*F*_c_^2^)/3
4260 reflections	(Δ/σ)_max_ = 0.001
237 parameters	Δρ_max_ = 0.35 e Å^−^^3^
0 restraints	Δρ_min_ = −0.40 e Å^−^^3^

### Special details

**Table:**

Geometry. All e.s.d.'s (except the e.s.d. in the dihedral angle between two l.s. planes) are estimated using the full covariance matrix. The cell e.s.d.'s are taken into account individually in the estimation of e.s.d.'s in distances, angles and torsion angles; correlations between e.s.d.'s in cell parameters are only used when they are defined by crystal symmetry. An approximate (isotropic) treatment of cell e.s.d.'s is used for estimating e.s.d.'s involving l.s. planes.
Refinement. Refinement of *F*^2^ against ALL reflections. The weighted *R*-factor *wR* and goodness of fit *S* are based on *F*^2^, conventional *R*-factors *R* are based on *F*, with *F* set to zero for negative *F*^2^. The threshold expression of *F*^2^ > σ(*F*^2^) is used only for calculating *R*-factors(gt) *etc*. and is not relevant to the choice of reflections for refinement. *R*-factors based on *F*^2^ are statistically about twice as large as those based on *F*, and *R*- factors based on ALL data will be even larger.

### Fractional atomic coordinates and isotropic or equivalent isotropic displacement parameters (Å^2^)

**Table:**

	*x*	*y*	*z*	*U*_iso_*/*U*_eq_	
S1	0.87241 (5)	0.20073 (3)	0.74789 (4)	0.04805 (14)	
O2	0.40215 (11)	−0.02903 (6)	0.69872 (10)	0.0400 (3)	
O3	0.83056 (11)	0.12613 (6)	0.65832 (10)	0.0381 (3)	
O1	0.21065 (13)	−0.09419 (7)	0.74469 (14)	0.0591 (4)	
O4	0.78108 (16)	0.19431 (11)	0.83435 (14)	0.0793 (5)	
O5	0.86864 (16)	0.27548 (8)	0.68331 (17)	0.0777 (5)	
C11	0.70098 (16)	−0.02353 (9)	0.74255 (14)	0.0371 (3)	
H11	0.6508	−0.0599	0.7837	0.045*	
C7	0.61790 (16)	0.04122 (9)	0.67270 (13)	0.0330 (3)	
C8	0.67779 (16)	0.11089 (9)	0.62748 (13)	0.0357 (3)	
C5	0.37380 (16)	0.09308 (9)	0.57881 (13)	0.0355 (3)	
C6	0.46285 (16)	0.03608 (9)	0.64762 (13)	0.0331 (3)	
C12	1.05418 (18)	0.17468 (10)	0.80513 (14)	0.0409 (4)	
C17	1.3108 (2)	0.20553 (11)	0.83220 (17)	0.0488 (4)	
H17	1.3876	0.2392	0.8175	0.059*	
C2	0.15966 (17)	0.02146 (10)	0.62033 (15)	0.0416 (4)	
H2	0.0578	0.0161	0.6127	0.050*	
C9	0.59404 (19)	0.16704 (11)	0.55527 (15)	0.0457 (4)	
H9	0.6394	0.2107	0.5234	0.055*	
C1	0.25173 (17)	−0.03775 (10)	0.69100 (16)	0.0417 (4)	
C4	0.11620 (19)	0.14562 (12)	0.49470 (15)	0.0484 (4)	
H4A	0.0150	0.1316	0.4958	0.073*	
H4B	0.1342	0.1451	0.4159	0.073*	
H4C	0.1363	0.1997	0.5272	0.073*	
C3	0.21464 (17)	0.08395 (10)	0.56514 (14)	0.0377 (3)	
C10	0.44325 (19)	0.15798 (11)	0.53077 (15)	0.0460 (4)	
H10	0.3870	0.1955	0.4817	0.055*	
C18	1.16766 (19)	0.22511 (11)	0.78238 (16)	0.0453 (4)	
H18	1.1480	0.2712	0.7346	0.054*	
C14	1.2259 (2)	0.08804 (12)	0.92495 (17)	0.0524 (4)	
H14	1.2454	0.0419	0.9725	0.063*	
C13	1.0825 (2)	0.10645 (12)	0.87741 (16)	0.0500 (4)	
H13	1.0054	0.0736	0.8935	0.060*	
C15	1.34283 (19)	0.13723 (11)	0.90328 (16)	0.0468 (4)	
C16	1.4988 (2)	0.11716 (14)	0.9572 (2)	0.0643 (5)	
H16A	1.5640	0.1583	0.9351	0.096*	
H16B	1.5251	0.0640	0.9306	0.096*	
H16C	1.5070	0.1164	1.0404	0.096*	
N1	0.83982 (14)	−0.03170 (8)	0.74886 (13)	0.0411 (3)	
N2	0.90695 (16)	−0.09370 (10)	0.81607 (14)	0.0519 (4)	
H2A	0.8559	−0.1258	0.8526	0.062*	
H2B	1.0006	−0.1009	0.8222	0.062*	

### Atomic displacement parameters (Å^2^)

**Table:**

	*U*^11^	*U*^22^	*U*^33^	*U*^12^	*U*^13^	*U*^23^
S1	0.0384 (2)	0.0402 (2)	0.0663 (3)	−0.00432 (16)	0.01104 (19)	−0.01826 (19)
O2	0.0273 (5)	0.0324 (5)	0.0604 (7)	0.0000 (4)	0.0079 (5)	0.0048 (5)
O3	0.0329 (5)	0.0358 (6)	0.0473 (6)	−0.0059 (4)	0.0112 (4)	−0.0073 (5)
O1	0.0357 (6)	0.0406 (7)	0.1039 (11)	−0.0001 (5)	0.0202 (7)	0.0154 (7)
O4	0.0507 (8)	0.1124 (13)	0.0803 (10)	−0.0143 (8)	0.0272 (8)	−0.0508 (10)
O5	0.0566 (9)	0.0329 (7)	0.1361 (15)	−0.0016 (6)	−0.0055 (9)	0.0022 (8)
C11	0.0309 (7)	0.0354 (8)	0.0459 (8)	−0.0009 (6)	0.0084 (6)	0.0008 (6)
C7	0.0311 (7)	0.0332 (7)	0.0355 (7)	−0.0003 (5)	0.0076 (6)	−0.0050 (6)
C8	0.0311 (7)	0.0372 (7)	0.0397 (8)	−0.0035 (6)	0.0084 (6)	−0.0034 (6)
C5	0.0339 (7)	0.0374 (8)	0.0352 (7)	0.0020 (6)	0.0052 (6)	−0.0027 (6)
C6	0.0322 (7)	0.0306 (7)	0.0374 (7)	−0.0006 (5)	0.0085 (6)	−0.0039 (6)
C12	0.0398 (8)	0.0394 (8)	0.0445 (8)	−0.0087 (6)	0.0099 (7)	−0.0108 (7)
C17	0.0415 (9)	0.0480 (9)	0.0580 (10)	−0.0150 (7)	0.0116 (8)	−0.0064 (8)
C2	0.0280 (7)	0.0410 (8)	0.0547 (10)	0.0021 (6)	0.0036 (7)	−0.0080 (7)
C9	0.0455 (9)	0.0460 (9)	0.0467 (9)	−0.0046 (7)	0.0107 (7)	0.0116 (7)
C1	0.0297 (7)	0.0334 (8)	0.0632 (10)	−0.0006 (6)	0.0113 (7)	−0.0047 (7)
C4	0.0412 (9)	0.0561 (10)	0.0459 (9)	0.0102 (8)	0.0012 (7)	0.0019 (8)
C3	0.0340 (7)	0.0396 (8)	0.0381 (8)	0.0050 (6)	0.0024 (6)	−0.0083 (6)
C10	0.0442 (9)	0.0485 (10)	0.0442 (9)	0.0033 (7)	0.0040 (7)	0.0109 (7)
C18	0.0466 (9)	0.0379 (8)	0.0518 (10)	−0.0106 (7)	0.0093 (7)	−0.0047 (7)
C14	0.0549 (11)	0.0502 (10)	0.0505 (10)	−0.0092 (8)	0.0040 (8)	0.0042 (8)
C13	0.0498 (10)	0.0508 (10)	0.0505 (10)	−0.0173 (8)	0.0116 (8)	−0.0010 (8)
C15	0.0433 (9)	0.0492 (10)	0.0481 (9)	−0.0063 (7)	0.0079 (7)	−0.0097 (7)
C16	0.0479 (11)	0.0686 (13)	0.0739 (14)	−0.0012 (9)	0.0030 (10)	−0.0027 (11)
N1	0.0321 (6)	0.0398 (7)	0.0513 (8)	0.0020 (5)	0.0070 (6)	0.0035 (6)
N2	0.0338 (7)	0.0548 (9)	0.0668 (10)	0.0064 (6)	0.0074 (7)	0.0177 (7)

### Geometric parameters (Å, °)

**Table:**

S1—O4	1.4197 (15)	C2—C3	1.342 (2)
S1—O5	1.4222 (16)	C2—C1	1.442 (2)
S1—O3	1.6003 (11)	C2—H2	0.9300
S1—C12	1.7441 (17)	C9—C10	1.376 (2)
O2—C6	1.3742 (18)	C9—H9	0.9300
O2—C1	1.3781 (18)	C4—C3	1.497 (2)
O3—C8	1.4119 (18)	C4—H4A	0.9600
O1—C1	1.203 (2)	C4—H4B	0.9600
C11—N1	1.2732 (19)	C4—H4C	0.9600
C11—C7	1.461 (2)	C10—H10	0.9300
C11—H11	0.9300	C18—H18	0.9300
C7—C8	1.397 (2)	C14—C13	1.374 (3)
C7—C6	1.409 (2)	C14—C15	1.395 (2)
C8—C9	1.382 (2)	C14—H14	0.9300
C5—C6	1.394 (2)	C13—H13	0.9300
C5—C10	1.395 (2)	C15—C16	1.501 (3)
C5—C3	1.453 (2)	C16—H16A	0.9600
C12—C18	1.385 (2)	C16—H16B	0.9600
C12—C13	1.387 (2)	C16—H16C	0.9600
C17—C15	1.383 (3)	N1—N2	1.3561 (19)
C17—C18	1.384 (3)	N2—H2A	0.8600
C17—H17	0.9300	N2—H2B	0.8600
			
O4—S1—O5	118.24 (11)	O1—C1—C2	126.59 (15)
O4—S1—O3	107.55 (8)	O2—C1—C2	117.10 (14)
O5—S1—O3	108.36 (9)	C3—C4—H4A	109.5
O4—S1—C12	110.73 (10)	C3—C4—H4B	109.5
O5—S1—C12	110.17 (9)	H4A—C4—H4B	109.5
O3—S1—C12	100.20 (7)	C3—C4—H4C	109.5
C6—O2—C1	121.77 (12)	H4A—C4—H4C	109.5
C8—O3—S1	114.70 (9)	H4B—C4—H4C	109.5
N1—C11—C7	122.14 (14)	C2—C3—C5	118.53 (14)
N1—C11—H11	118.9	C2—C3—C4	121.58 (15)
C7—C11—H11	118.9	C5—C3—C4	119.84 (15)
C8—C7—C6	114.76 (13)	C9—C10—C5	120.70 (15)
C8—C7—C11	125.94 (13)	C9—C10—H10	119.7
C6—C7—C11	119.30 (13)	C5—C10—H10	119.7
C9—C8—C7	123.11 (14)	C17—C18—C12	118.61 (17)
C9—C8—O3	117.97 (13)	C17—C18—H18	120.7
C7—C8—O3	118.91 (13)	C12—C18—H18	120.7
C6—C5—C10	117.71 (14)	C13—C14—C15	121.31 (18)
C6—C5—C3	118.63 (14)	C13—C14—H14	119.3
C10—C5—C3	123.60 (14)	C15—C14—H14	119.3
O2—C6—C5	120.98 (13)	C14—C13—C12	119.15 (16)
O2—C6—C7	115.20 (13)	C14—C13—H13	120.4
C5—C6—C7	123.80 (14)	C12—C13—H13	120.4
C18—C12—C13	121.00 (16)	C17—C15—C14	118.17 (17)
C18—C12—S1	119.25 (14)	C17—C15—C16	121.05 (17)
C13—C12—S1	119.67 (13)	C14—C15—C16	120.78 (18)
C15—C17—C18	121.75 (16)	C15—C16—H16A	109.5
C15—C17—H17	119.1	C15—C16—H16B	109.5
C18—C17—H17	119.1	H16A—C16—H16B	109.5
C3—C2—C1	122.85 (14)	C15—C16—H16C	109.5
C3—C2—H2	118.6	H16A—C16—H16C	109.5
C1—C2—H2	118.6	H16B—C16—H16C	109.5
C10—C9—C8	119.70 (15)	C11—N1—N2	117.71 (14)
C10—C9—H9	120.2	N1—N2—H2A	120.0
C8—C9—H9	120.2	N1—N2—H2B	120.0
O1—C1—O2	116.30 (15)	H2A—N2—H2B	120.0
			
O4—S1—O3—C8	42.38 (14)	C7—C8—C9—C10	3.9 (3)
O5—S1—O3—C8	−86.51 (12)	O3—C8—C9—C10	−174.80 (15)
C12—S1—O3—C8	158.10 (11)	C6—O2—C1—O1	−175.70 (15)
N1—C11—C7—C8	−15.2 (2)	C6—O2—C1—C2	4.0 (2)
N1—C11—C7—C6	165.64 (15)	C3—C2—C1—O1	178.45 (18)
C6—C7—C8—C9	−5.4 (2)	C3—C2—C1—O2	−1.2 (2)
C11—C7—C8—C9	175.39 (15)	C1—C2—C3—C5	−1.0 (2)
C6—C7—C8—O3	173.29 (13)	C1—C2—C3—C4	−178.52 (15)
C11—C7—C8—O3	−5.9 (2)	C6—C5—C3—C2	0.7 (2)
S1—O3—C8—C9	72.10 (16)	C10—C5—C3—C2	−176.51 (16)
S1—O3—C8—C7	−106.67 (13)	C6—C5—C3—C4	178.20 (14)
C1—O2—C6—C5	−4.5 (2)	C10—C5—C3—C4	1.0 (2)
C1—O2—C6—C7	173.91 (13)	C8—C9—C10—C5	0.5 (3)
C10—C5—C6—O2	179.38 (14)	C6—C5—C10—C9	−2.9 (2)
C3—C5—C6—O2	2.0 (2)	C3—C5—C10—C9	174.32 (16)
C10—C5—C6—C7	1.1 (2)	C15—C17—C18—C12	0.2 (3)
C3—C5—C6—C7	−176.24 (14)	C13—C12—C18—C17	0.8 (3)
C8—C7—C6—O2	−175.49 (13)	S1—C12—C18—C17	177.42 (13)
C11—C7—C6—O2	3.8 (2)	C15—C14—C13—C12	1.0 (3)
C8—C7—C6—C5	2.9 (2)	C18—C12—C13—C14	−1.3 (3)
C11—C7—C6—C5	−177.88 (14)	S1—C12—C13—C14	−177.96 (14)
O4—S1—C12—C18	−133.39 (14)	C18—C17—C15—C14	−0.5 (3)
O5—S1—C12—C18	−0.71 (17)	C18—C17—C15—C16	−179.64 (18)
O3—S1—C12—C18	113.30 (14)	C13—C14—C15—C17	0.0 (3)
O4—S1—C12—C13	43.30 (17)	C13—C14—C15—C16	179.07 (19)
O5—S1—C12—C13	175.97 (14)	C7—C11—N1—N2	179.96 (14)
O3—S1—C12—C13	−70.01 (14)		

### Hydrogen-bond geometry (Å, °)

**Table:**

*D*—H···*A*	*D*—H	H···*A*	*D*···*A*	*D*—H···*A*
N2—H2A···O5^i^	0.86	2.59	3.303 (2)	141
N2—H2B···O1^ii^	0.86	2.27	3.045 (2)	150
C10—H10···O4^iii^	0.93	2.55	3.469 (2)	169
C16—H16A···O4^ii^	0.96	2.55	3.405 (3)	149
C18—H18···O1^iv^	0.93	2.53	3.166 (2)	126

Symmetry codes: (i) −*x*+3/2, *y*−1/2, −*z*+3/2; (ii) *x*+1, *y*, *z*; (iii) *x*−1/2, −*y*+1/2, *z*−1/2; (iv) −*x*+3/2, *y*+1/2, −*z*+3/2.
